# Supplementary material for: The Systems Biology Research Tool: evolvable open-source software
Source: BMC Syst Biol. 2008 Jun 29;2:55. doi: 10.1186/1752-0509-2-55 (PMC2446383; doi:10.1186/1752-0509-2-55)
Supplement: Additional file 1 — SBRT Archive. An archive of the current version of the Systems Biology Research Tool. [file 1752-0509-2-55-S1.zip › sbrt-1.4.0/doc/developers_guide/api/sbrt/kernel/fba/comp/FluxCapComputer.html]

FluxCapComputer


|  |  |  |
| --- | --- | --- |
| **Overview** \| **Package** \| **Class** \| **Use** \| **Tree** \| **Deprecated** \| **Index** \| **Help** | |  |
| **PREV CLASS**   **NEXT CLASS** | **FRAMES**    **NO FRAMES**     **All Classes** |  |
| SUMMARY: NESTED \| FIELD \| CONSTR \| METHOD | DETAIL: FIELD \| CONSTR \| METHOD |  |


---


## sbrt.kernel.fba.comp Class FluxCapComputer

```
java.lang.Object
  sbrt.kernel.fba.comp.FluxCapComputer
```

---

``` public class FluxCapComputer extends java.lang.Object ```

This class is used to compute *flux caps* for use in Mahadevan-Schilling
flux interval computations. A flux cap is a linear combination of fluxes
that can be used to cap, or bound, a naturally unbound flux.

**Author:**
:   This class was written and documented by Jeremiah Wright
    while in the Wagner lab.

---

**Constructor Summary**

`FluxCapComputer(Fluxome fluxome,
java.util.Collection<LinearComb<java.lang.String>> allCycles)`
  
          Constructs a new flux cap computer from the cycles contained
in the provided fluxome.

`FluxCapComputer(Fluxome fluxome,
DoubleMatrix<?,java.lang.String> cycleMatrix)`
  
          Constructs a new flux cap computer from the cycles contained
in the provided fluxome.


| **Method Summary** | |
| --- | --- |
| `java.util.Map<java.lang.String,java.util.Set<LinearComb<java.lang.String>>>` | `getFluxCaps()`             Computes and returns all flux caps. |
| `Fluxome` | `getFluxome()`             Returns the fluxome for which flux caps are computed. |
| `ProgressMonitor` | `getProgress()`             Returns a progress monitor for this flux cap computer. |

**Methods inherited from class java.lang.Object**

`clone, equals, finalize, getClass, hashCode, notify, notifyAll, toString, wait, wait, wait`

**Constructor Detail**

### FluxCapComputer

```
public FluxCapComputer(Fluxome fluxome,
                       DoubleMatrix<?,java.lang.String> cycleMatrix)
```

:   Constructs a new flux cap computer from the cycles contained
    in the provided fluxome.

    **Parameters:**: `fluxome` - the fluxome for which flux caps will be computed.: `cycleMatrix` - the collection of all cycles in the provided fluxome.

---


### FluxCapComputer

```
public FluxCapComputer(Fluxome fluxome,
                       java.util.Collection<LinearComb<java.lang.String>> allCycles)
```

:   Constructs a new flux cap computer from the cycles contained
    in the provided fluxome.

    **Parameters:**: `fluxome` - the fluxome for which flux caps will be computed.: `allCycles` - the collection of all cycles in the provided fluxome as linear combinations.


**Method Detail**

### getProgress

```
public ProgressMonitor getProgress()
```

:   Returns a progress monitor for this flux cap computer.

    :   **Returns:**: a progress monitor for this flux cap computer.

---


### getFluxome

```
public Fluxome getFluxome()
```

:   Returns the fluxome for which flux caps are computed.

    :   **Returns:**: the fluxome for which flux caps are computed.

---


### getFluxCaps

```
public java.util.Map<java.lang.String,java.util.Set<LinearComb<java.lang.String>>> getFluxCaps()
```

:   Computes and returns all flux caps.

    :   **Returns:**: reaction names mappes to their corresponding set of flux caps.


---


|  |  |  |
| --- | --- | --- |
| **Overview** \| **Package** \| **Class** \| **Use** \| **Tree** \| **Deprecated** \| **Index** \| **Help** | |  |
| **PREV CLASS**   **NEXT CLASS** | **FRAMES**    **NO FRAMES**     **All Classes** |  |
| SUMMARY: NESTED \| FIELD \| CONSTR \| METHOD | DETAIL: FIELD \| CONSTR \| METHOD |  |


---
